# The inversion effect on the cubic humanness-uncanniness relation in humanlike agents

**DOI:** 10.3389/fpsyg.2023.1222279

**Published:** 2023-08-29

**Authors:** Alexander Diel, Wataru Sato, Chun-Ting Hsu, Takashi Minato

**Affiliations:** ^1^Guardian Robot Project, RIKEN, Kyoto, Japan; ^2^Cardiff University School of Psychology, Cardiff University, Cardiff, United Kingdom

**Keywords:** configural processing, dynamic facial expression, emotion expression, inversion effect, uncanny valley

## Abstract

The uncanny valley describes the typically nonlinear relation between the esthetic appeal of artificial entities and their human likeness. The effect has been attributed to specialized (configural) processing that increases sensitivity to deviations from human norms. We investigate this effect in computer-generated, humanlike android and human faces using dynamic facial expressions. Angry and happy expressions with varying degrees of synchrony were presented upright and inverted and rated on their eeriness, strangeness, and human likeness. A sigmoidal function of human likeness and uncanniness (“uncanny slope”) was found for upright expressions and a linear relation for inverted faces. While the function is not indicative of an uncanny valley, the results support the view that configural processing moderates the effect of human likeness on uncanniness and extend its role to dynamic facial expressions.

## The uncanny valley in human–robot interaction

Humanlike robots and androids are being increasingly used in society. Social robots provide an alternative to human caretakers for older adults to mitigate loneliness or as companions for children with autism spectrum disorder (Adams and Robinson, 2011; Kumazaki et al., 2017; Fattal et al., 2020). However, robotic assistance may encounter barriers to acceptance, especially in tasks typically requiring human–human interaction, such as personal care or leisure activities (Smarr et al., 2014). One potential barrier is the uncanny valley effect, in which individuals feel an aversion to artificial entities that closely resemble humans (Mori et al., 2012). Research on the uncanny valley typically relies on self-report measures of human likeness or realism for the independent variable and affect measures for the dependent variable, typically likability, eeriness, or a related measure (Diel et al., 2022). Human likeness is a multidimensional construct describing an entity’s closeness to human norms in outward appearance, behavior, or inner states, although these components tend to be unspecified or combined in self-reported human likeness measures to a holistic perception of humanness (Mori et al., 2012; von Zitzewitz et al., 2013; Ho and MacDorman, 2017; Diel et al., 2022). Uncanniness is considered a specific negative experience associated with emotions like fear, anxiety, and disgust, is potentially linked to the inability to explain presented information, and is considered a specific component of the uncanny valley (Ho et al., 2008; MacDorman and Entezari, 2015; Mangan, 2015; Diel et al., 2022).

Many empirical studies have investigated the relation between human likeliness and emotional responses to humanlike artificial entities. Although the results are somewhat inconsistent across studies (Diel et al., 2022), a recent meta-analysis analyzed the data of 49 studies testing the relation between human likeness and likeability to robot agents (Mara et al., 2022). Researchers found the relation between multiple artificial entities’ human likeness and likability was a cubic (sigmoidal) function. The results indicate that the relation between the human likeness and emotional impressions of artificial entities takes on a nonlinear shape, which could be associated with the uncanny valley phenomenon.

However, uncertainties remain on whether there is a sigmoidal relation between likability and anthropomorphism. First, the meta-analysis by Mara et al. (2022) focused on the Godspeed likability scales (Bartneck et al., 2009) and non-realistic humanlike robots (e.g., NAO). In contrast, uncanny valley research typically assesses the uncanniness of more realistic robots, computer-generated (CG) characters, and fully human stimuli (Diel et al., 2022).

While multiple psychological mechanisms underlying the cubic relation between human likeness and emotional impressions have been proposed and investigated, there is little consensus on the exact processes (Wang et al., 2015; Reuten et al., 2018; Kätsyri et al., 2019; Zhang et al., 2020; Diel and MacDorman, 2021). Categorization difficulty or ambiguity has been proposed to cause uncanniness in entities lying at the borders between human and robot categories (Yamada et al., 2013; Cheetham et al., 2014). Evolutionarily, deviating features in an otherwise humanlike entity may elicit disease avoidance mechanisms (MacDorman and Entezari, 2015). Anomalies or deviations in human norms may also elicit error signals in predictive coding (Saygin et al., 2012). On a perceptual level, mismatching or atypical features may appear eerie or uncanny, especially in more realistic and human entities (MacDorman et al., 2009; Kätsyri et al., 2019; Diel and MacDorman, 2021).

This study focuses on the last explanation. We hypothesize that the candidate mechanisms include the configural processing of faces and facial expressions. The uncanny valley effect has been attributed to the specialized processing of familiar categories resulting in stronger error signals for artificial entities (MacDorman et al., 2009; Chattopadhyay and MacDorman, 2016; Kätsyri et al., 2019; Diel and MacDorman, 2021; Diel and Lewis, 2022). Specialized processing describes a particular type of domain-specific cognitive processing marked by an improved ability to discriminate or recognize individual exemplars and by the recruitment of specialized neural areas (Tanaka and Farah, 1993; Kanwisher, 2000; Carbon and Leder, 2006). Atypicalities or deviations may induce negative esthetic evaluations, especially sensitive for stimulus categories that elicit specialized processing.

Configural processing is a form of specialized processing, and the configural processing of faces depends on their upright orientation. The inversion of faces disrupts this processing (*inversion effect*; Kanwisher and Moscovitch, 2000; Carbon and Leder, 2006). Configural processing also improves the processing of facial expressions and is disrupted when expressions are inverted (Ambadar et al., 2005; Bould and Morris, 2008; Tobin et al., 2016). Facial esthetic ratings’ variance decreases when faces are inverted, likely because face processing becomes less accurate (Bäuml, 1994; Santos and Young, 2008; Leder et al., 2017). Furthermore, uncanniness ratings of faces are less severe when faces are inverted due to a decreased ability to detect changes or distortions in a face (Diel and Lewis, 2022). However, neither the effect of inversion on the likability of entities varying in human likeness nor the uncanniness of dynamic facial expressions has been investigated. As specialized processing is more pronounced in more realistic faces (Crookes et al., 2015), inversion may disrupt more subtle differences in esthetic ratings of highly realistic dynamic expressions. This effect of specialized processing on dynamic face processing may explain why subtle facial movements in realistic androids may appear eerie or uncanny.

The processing of dynamic emotion expressions has been well-investigated using virtual (computer-generated) agents whose temporal trajectory of face muscle movement can be easily manipulated and controlled (Krumhuber et al., 2012; Pan and Hamilton, 2018). Although virtual agents are not physically present when interacting with a human, they offer a way to study the effects of specialized processing in the uncanniness of emotion expressions alongside android stimuli.

This work aims to investigate whether the relation between human likeness and uncanniness is cubic for humanlike agents and whether the relation is mediated by configural processing. To test the humanlike agents, we presented dynamic emotional expressions of human, android, and CG faces. To test the effect of configural processing of faces, we compared upright and inverted facial stimuli. We presented the various types of facial stimuli by using the emotional facial expressions of negative and positive valence (i.e., anger and happiness) and presenting facial expressions with different facial action patterns over time, which were shown to elicit slightly different emotional impressions. Following previous meta-analyses (Diel et al., 2022; Mara et al., 2022), the uncanny valley effect is investigated by testing for a cubic function between ratings of esthetics and human likeness:

*Hypothesis 1*. A cubic function relates uncanniness to human likeness in upright facial expressions.

However, as we propose that the nonlinear relation between uncanniness and human likeness results from specialized processing of faces and facial expressions, this effect should not occur when the expressions are presented inverted:

*Hypothesis 2*. A linear function relates uncanniness to human likeness in inverted facial expressions.

However, if upright and inverted facial expressions produced the same uncanniness function, this would indicate a lack of inversion effect, suggesting that specialized processing plays little to no role in evaluating the esthetics of artificial entities.

## Methods

### Participants

Sixty-four Japanese volunteers participated in this study (31 females, 31 males, and two who preferred not to specify their gender; mean ± SD age, 30.65 ± 3.88 years). The required sample size was determined using an *a priori* power analysis using G*Power software ver. 3.1.9.2 (Faul et al., 2007). As an approximation of the present analysis using linear mixed-effects models containing seven dependent variables (i.e., the interaction model), a multiple linear regression model with seven dependent variables was analyzed. A power analysis for the coefficient evaluation (two-tailed) with the assumption of f^2^ of 0.15 (medium size effect), α level of 0.05, and power (1–β) of 0.80 showed that 55 participants were needed.

Because G*Power may be insufficient for power analysis of linear mixed models due to its inability to handle random effects, an additional, simulation-based power analysis was conducted using the simr R package (Green and MacLeod, 2016) and linear mixed model analyses using the lme4 package including random slopes (Bates et al., 2015). Power analysis was conducted using a pilot sample of *n* = 11 assuming the same coefficients, an α level of 0.05, and 100 simulation runs. For a power of 80%, for linear, quadratic, and cubic human likeness terms respectively, sample sizes of 7, 16, and 14 were necessary. At the *n* of 64, a power of 100% was reached. The second power analysis thus suggests that no larger sample size would be needed.

Participants were recruited through web advertisements distributed via CrowdWorks (Tokyo, Japan). After the procedures had been explained, all participants provided written informed consent to participate in the study, which the Ethics Committee of RIKEN approved. The experiment was performed in accordance with the Declaration of Helsinki.

### Materials

#### Actors

##### Android

The study used the android Nikola. Nikola’s 35 pneumatic actuators reproduce the facial actions required to express six basic human emotions (Sato et al., 2022). Their temporal resolution of milliseconds enables natural emotion expression. Android videos were created by filming Nikola’s frontal emotion expressions.

#### Human

Human videos were created using angry and happy expressions from the AIST Facial Expression Database (Fujimura and Umemura, 2018).

##### CG

CG videos were created using FACSGen (Roesch et al., 2011; Krumhuber et al., 2012).

#### Videos

For android and CG faces, the following face action units (AUs) were used for the expressions: angry: 4 (brow lowerer), 5 (upper lid raiser), 7 (lid tightener), 23 (lip tightener); happy: 6 (cheek raiser), 12 (lip corner puller). Asynchronous motion was created for android videos by delaying motion onset. Asynchronies were either absent (original video or synchronous motion), delayed (the movement of the upper right half of the face was delayed 250 ms and the upper left half 500 ms), or doubly delayed (the upper right half of the face was delayed 500 ms and the upper left half 1,000 ms). The lower half of the face started to move at the same time in each condition. Asynchronous motion was created for CG and human videos by delaying motion onset using Adobe Premiere.

All videos were edited to have the noses of each actor at the same height, to cut off at the neck (bottom), head (top), and ears (left and right), and to show a white background. All videos were 1.25 s long and depicted the onset of one out of two emotion expressions: angry and happy.

**Figure 1:**
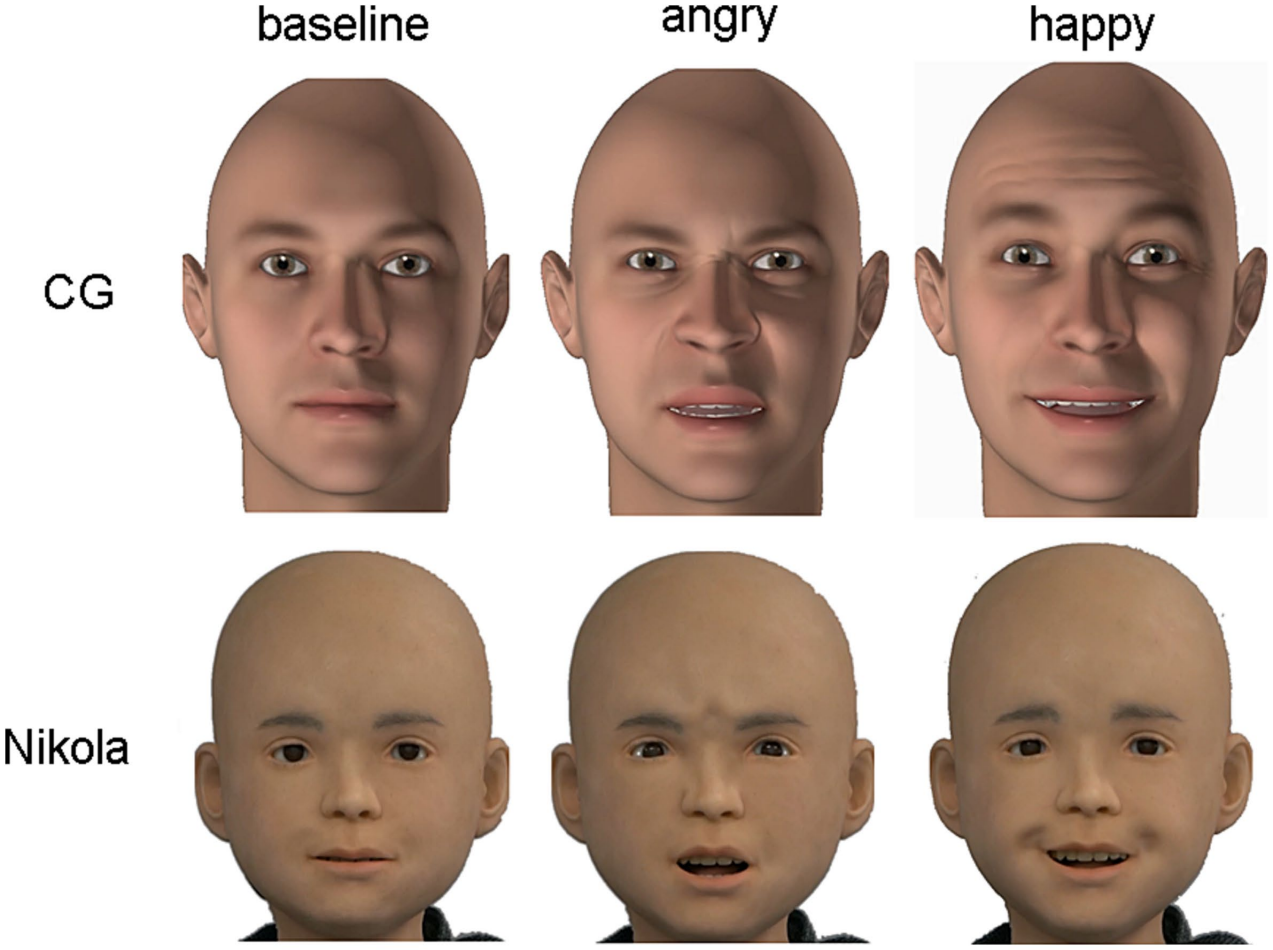
CG (computer-generated; top) and android (bottom) stimuli across emotion conditions. Baseline (neutral) expressions are to the right, followed by angry and happy expressions.

A total of 36 videos (3 actors, 3 asynchrony levels, 2 orientations, 2 emotions) were used. Screenshots of the android and CG expressions are depicted in Figure 1. The android and CG stimuli are available in the Supplementary material. AIST prohibits the distribution of human stimuli.

#### Stimulus validation

A stimulus validation pilot study was conducted to test whether the actors’ objective and subjective emotional expressions differed.

##### Objective expressions

For validation of objective expressions, facial movements of the base stimuli of angry and happy expressions for each actor were analyzed using OpenFace (version 2.2.0; Baltrusaitis et al., 2018). Face action units (AUs) characteristic of angry and happy emotion expressions were used as indicators.

**Figure 2:**
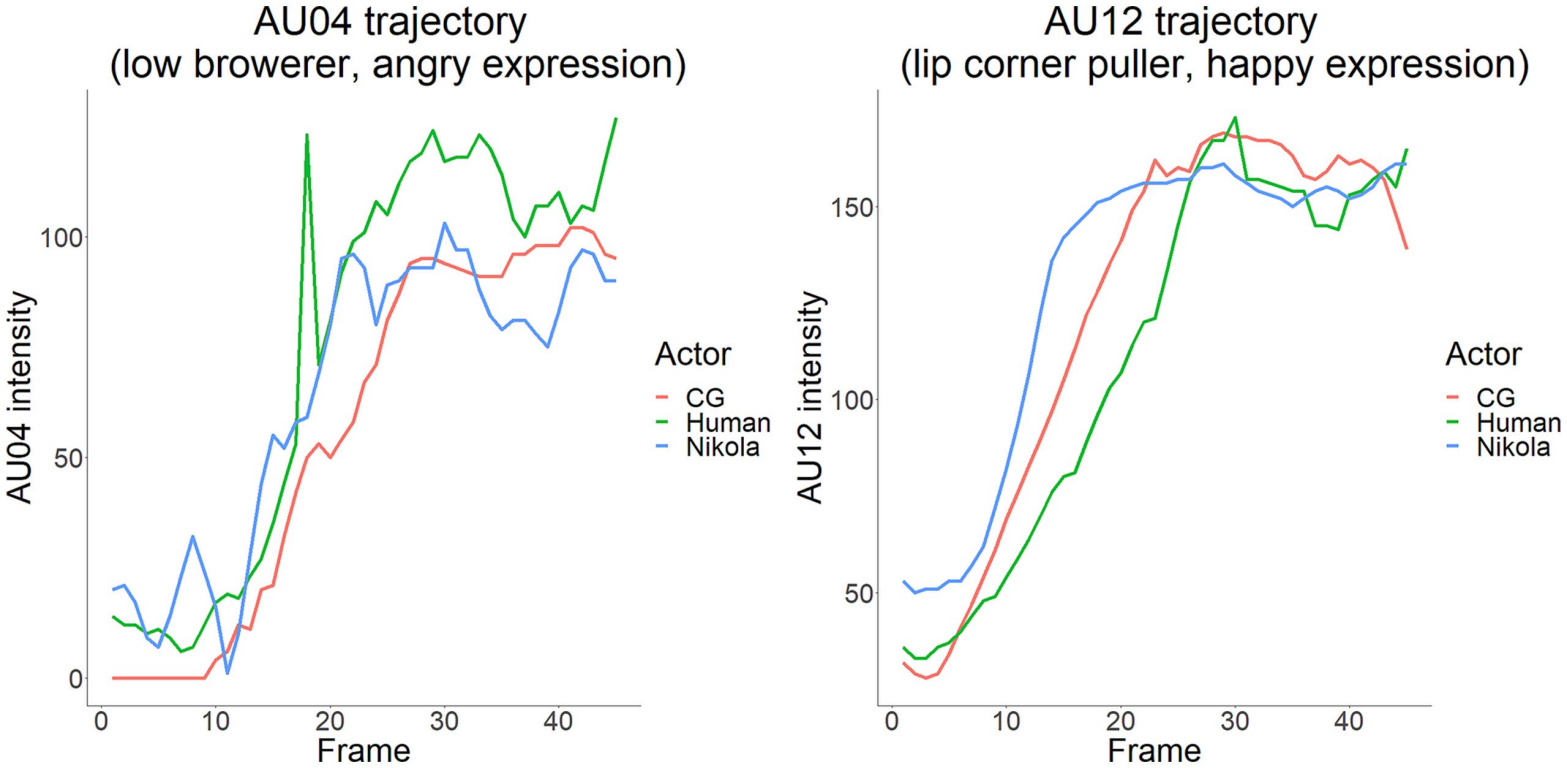
The intensity of face action units AU4 and AU12 across actor types. Values were analyzed automatically using OpenFace. CG = computer-generated.

Specifically, AU4 (brow lowerer) and AU12 (lip corner puller) values were used to indicate angry and happy expressions, respectively. The trajectories of both AUs are depicted in Figure 2. While Nikola’s AU12 trajectories began to increase earlier than the other actors, there were no strong deviations in AU intensity between the three actors, indicating that the intensity of AU expressions is analogous across actors.

##### Subjective expressions

For subjective expressions, a questionnaire study has been conducted. Single-scale items of valence and arousal based on the bipolar valence-arousal modes were used to assess emotional expressions. Eleven participants were asked to rate the faces on the following scales ranging from 0 to 100: how angry the face is, how happy the face is, emotional arousal, and emotional valence. The study was conducted online. Results show no significant main effects of actor type on ratings on how happy (*F*(2.63) = 0.1, *p* = 0.93) or angry (*F*(2.63) = 0.3, *p* = 0.76) the faces were, neither on arousal (*F*(2.63) = 0.1, *p* = 0.95) or valence (*F*(2.63) = 0.1, *p* = 0.89) ratings.

Thus, for both emotions, indicators for both objective and subjective intensity of emotional expressions did not differ across actors.

### Procedure

The experiment was conducted online. After providing informed consent, participants were linked to the experiment page. There, participants were shown each video in a randomized order. Participants had to rate each video on three scales used in a previous study (Diel et al., 2022): *uncanny*, *strange*, and *humanlike*. Specifically, participants were shown the terms and had to rate the video on uncanny/strange/humanlike scales ranging from 0 to 100. There was no time limit on rating the videos, which could be repeated at any time.

### Statistical analysis

Linear mixed-effect models were used for data analysis. Models were constructed using uncanny ratings as the dependent variable. The main effect model included linear, quadratic, and cubic functions of human likeness and orientation as independent variables, and the interaction model included linear, quadratic, and cubic functions of human likeness, orientation, and interactions between each function of human likeness and orientation as independent variables. Random by-participant intercepts were used for each model. Random by-participant intercepts were added as per traditional repeated-measures analyses; in addition, our preliminary analysis for the interaction model indicated that model comparison using Akaike information criterion (AIC) preferred the model with only by-participant intercepts compared with that with by-participant intercepts and slopes (AIC = 20,280 vs. 20,294).

All statistical analyses were performed using the statistics and machine learning toolbox in MATLAB 2020a (MathWorks, Natick, MA, United States). The relations between uncanny and humanlike ratings were analyzed according to the study’s purpose. The data, stimuli (except human videos), and analysis are available at https://osf.io/9cmhp.

## Results

**Figure 3:**
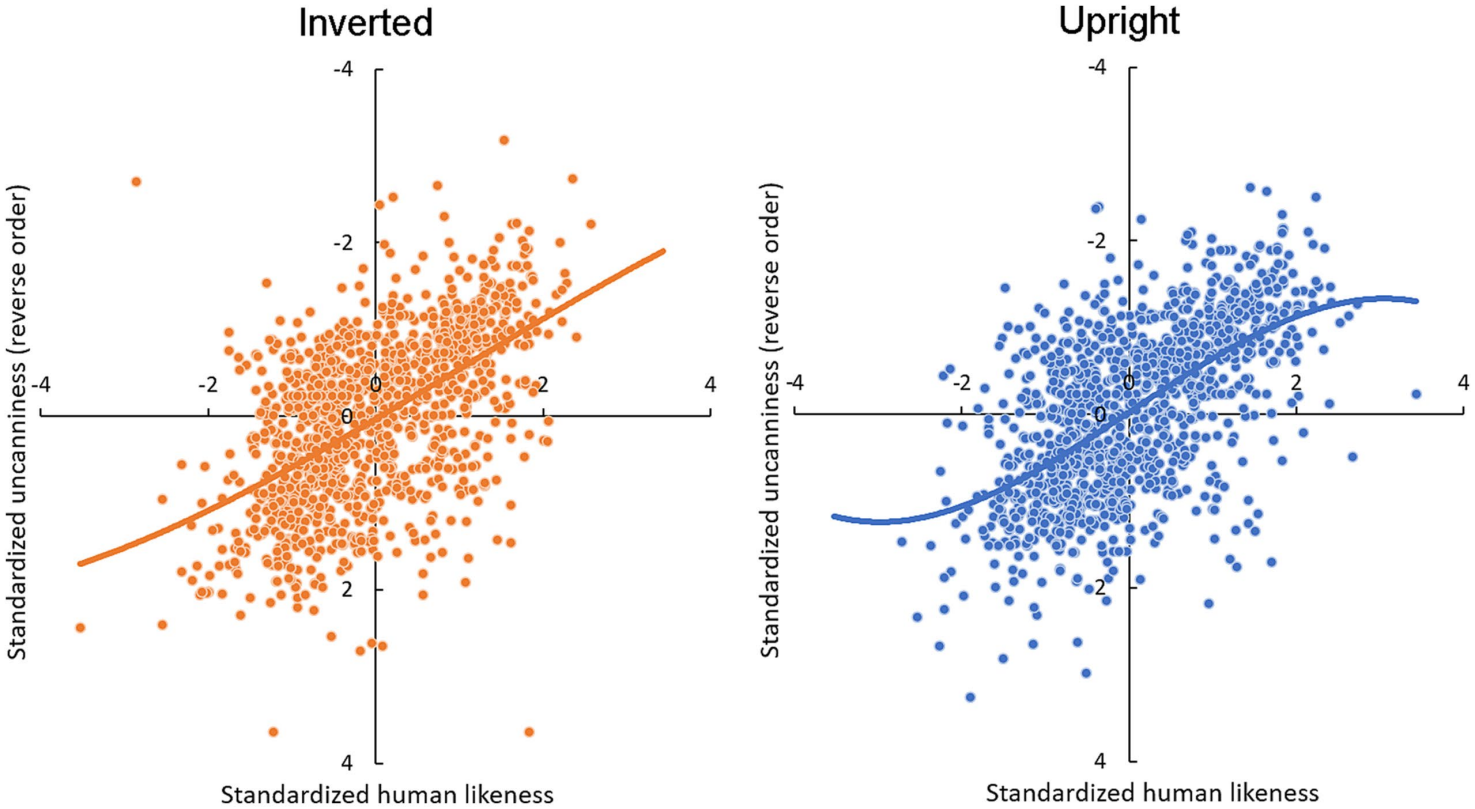
Scatter plots and cubic regression lines between humanlike and uncanny ratings for upright and inverted presented faces. Standardized scores are shown to indicate consistent patterns across participants. Points represent raw data points.

**Figure 4:**
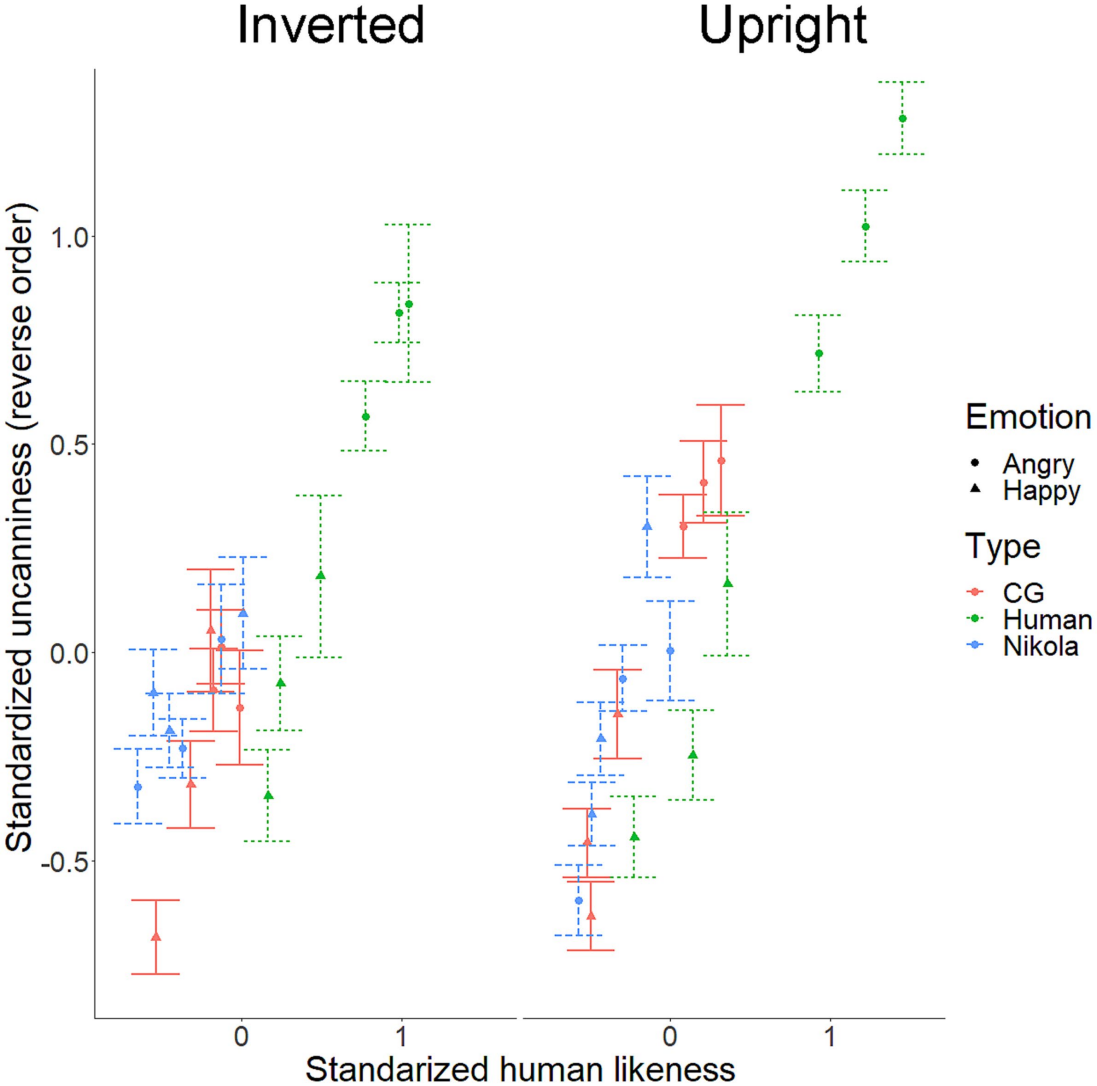
Average uncanniness ratings for each stimulus along the human likeness axis, divided by orientation. Color and point shape indicate agent and emotion type. Error bars indicate standard errors. CG = computer-generated face created via FACSGen.

All scales were transformed using z-standardization. Z-transformed *strange* and *uncanny* items were combined into an *uncanniness* index by calculating trial-based averages with internal consistency (Cronbach’s alpha) of *α* = 0.9. The relations between uncanny and humanlike ratings are plotted in Figure 3. Average uncanniness and human likeness values across stimuli are depicted in Figure 4.

Model comparison using AIC supported the interaction model (main effect vs. interaction: 20,282 vs. 20,280). Evaluation of beta estimates using Satterthwaite’s approximation for the interaction model revealed that cubic human likeness × orientation was significant (*F*(1, 2236.1)= 7.0, *p* = 0.008). In addition, linear human likeness (*F*(1, 2247.4) = 170.9, *p* < 0.001) and the interaction between linear human likeness and orientation (*F*(1, 2238.8) = 5.7, *p* = 0.017) were significant.

Follow-up analyses were conducted for each orientation condition using the simple main model, including linear, quadratic, and cubic functions of human likeness as independent variables. For upright faces, linear (*F*(1, 1090.4) = 255.2, *p* < 0.001) and cubic (*F*(1, 1100.0) = 4.6, *p* = 0.033) function of human likeness were significant. For inverted faces, only linear human likeness was significant (*F*(1, 1114.2) = 162.4, *p* < 0.001); the cubic function did not reach significance (*F*(1, 1124.1) = 0.6, *p* = 0.459).

In addition, AIC-based model comparisons between cubic and linear models for each orientation supported the cubic model for the upright condition (linear vs. cubic: 10,110 vs. 10,109) and the linear model for the inverted condition (linear vs. cubic: 10,286 vs. 10,290). Thus, hypotheses 1 and 2 were supported.

To investigate the modulatory effects of agent (human, android, and CG) and emotion (anger and happiness), we constructed a linear mixed-effect model adding these effects to the interaction model. Specifically, independent variables included human likeness functions (linear, quadratic, and cubic), orientation, agent, and emotion, and their two-, three-, and four-way interactions. The results showed that cubic human likeness x orientation interaction was significant (*F*(1, 2236.1) = 4.5, *p* = 0.034) and that no significant higher-order interactions were related to this two-way interaction. However, cubic human likeness × orientation × agent interaction reached a non-significant trend (cubic human likeness × orientation × agent: *F*(2, 2237.8) = 2.4, *p* = 0.087; cubic human likeness × orientation × emotion: *F*(1, 2236.7) < 0.1, *p* = 0.853; cubic human likeness × orientation × agent × emotion: *F*(2, 2236.4) = 0.6, *p* = 0.546). The results suggest that the effects of agent and emotion are not evident in the above results.

In summary, a cubic (increasing sigmoid) relation between uncanniness and human likeness was observed for upright, but not inverted, expressions. The results support the view that (1) relations between human likeness and uncanniness ratings for human and humanlike agents’ expressions are cubic and that (2) these relations result from configural processing.

## Discussion

The present research investigated the effect of inversion, a proxy of configural processing in faces and facial expressions, on the uncanniness of different agents’ facial expressions across human likeness. Differences between upright and inverted expressions were found. Specifically, a cubic (increasing sigmoid) function of human likeness best explained the uncanniness of facial expressions, consistent with previous research (Diel et al., 2022; Mara et al., 2022). Meanwhile, only a linear function of human likeness was significant for inverted facial expressions. Thus, a characteristic cubic, in this case, a sigmoid function of human likeness on esthetic appeal, is only present when the configural processing of facial expressions remains intact. This suggests that the typical observations on the relation between artificial agents’ esthetic ratings and human likeness depend on specialized processing mechanisms.

The present study did not find a proper uncanny valley because uncanniness ratings increased monotonically (Mori et al., 2012). A limited stimulus range has been suggested to be one cause of failing to find an uncanny valley function (Diel et al., 2022): uncanny valley functions are observed when stimuli range from less realistic (mechanically robotic or cartoon) to realistic human faces (Mathur et al., 2020). CG and android faces used here may have been too realistic to plot a complete uncanny valley function. Similarly, previous research observed linear functions using only a limited stimulus range lacking less realistic stimuli (Kätsyri et al., 2019).Selecting additional less humanlike robot stimuli may have led to a proper uncanny valley. However, manipulation of emotion expressions is difficult in robot faces, which would thus not have been suitable for this study. Similarly, although sigmoidal relations between human likeness and likability were found in a previous meta-analysis (Mara et al., 2022), these were found when including studies lacking full human stimuli. For research that includes a broader range of stimuli varying on human likeness, cubic functions akin to Mori et al.’s (2012) uncanny valley are expected (Diel et al., 2022). Thus, the exact cause of the sigmoidal uncanniness function remains unclear. MacDorman and Chattopadhyay (2016) proposed that this nonlinear relation may result from a higher sensitivity to deviations in more familiar face categories, which Kätsyri et al. (2019) identified with the “uncanny slope” found in their results. Consistently, as inversion reduces this sensitivity (Diel and Lewis, 2022), an “uncanny slope” was not found for inverted expressions in the present study, as participants were less sensitive to deviations in inverted stimuli.

Furthermore, logistic patterns akin to two levels connected by an increasing slope are also found in categorization tasks plotted against human likeness (Looser and Wheatley, 2010; Cheetham et al., 2011; MacDorman and Chattopadhyay, 2016). Thus, categorization as human or nonhuman may determine affect ratings, which inversion may influence.

Nevertheless, the results show that configural processing moderates the effect of human likeness on uncanniness. Specialized processing may act as a gateway to enhanced detection of errors or deviations, which may lead to negative evaluations (Chattopadhyay and MacDorman, 2016; Diel and Lewis, 2022). Accordingly, ratings of facial esthetics are more sensitive when faces are presented upright instead of inverted (Bäuml, 1994; Santos and Young, 2008; Leder et al., 2017). The present results show for the first time that configural processing’s role in esthetics extends beyond facial structure to include dynamic facial expressions. Furthermore, the results indicate specialized processing plays a role in evaluating artificial entities (e.g., Diel and MacDorman, 2021) and their facial expressions.

Social robots with the ability to emulate human emotion expressions and affect have the potential to provide emotional support and experiences of social bonding and connectedness (Kirby et al., 2010; Sato et al., 2022). The processing of artificial entities’ emotional expressions may fall under similar scrutiny as evaluations of their physical appearance, leading to perceptions of uncanniness. Configural processing may sensitize the uncanniness of artificial entities expressing emotions with their face, in line with previous research showing analogous effects with faces (Diel and Lewis, 2022). As inversion effects increase with an entity’s level of realism (Crookes et al., 2015), designing social robots in a highly realistic or humanlike manner may increase the chance that imperfections in their appearance or face motion are detected and negatively evaluated. Instead, the design of social robots may profit from less realistic, stylistic, or cartoon-like designs that do not recruit specialized processing mechanisms sensitized to uncanniness. Alternatively, care can be taken for the design of realistic artificial entities, like social robots or CG animations, not only in their appearance but also in the temporal aspects of facial expressions.

In this study, participants were allowed to watch the stimulus videos repeatedly. While this ensures more accurate emotion expression processing, repeated exposure may have decreased uncanniness ratings through habituation. In addition, human stimuli in this study possessed human hair while the android and CG stimuli did not, which could confound human likeness and uncanniness measures. Future research may replicate results with shorter video exposure and controlled stimulus appearance. In addition, future research may investigate a broader range of actors varying in human likeness, including low-realism actors, to attempt to replicate a full uncanny valley function. Finally, perceptual specialization could be measured more directly in future research, for example, by adding an inversion recognition task.

## Conclusion

The statistical relation between human likeness and esthetic appeal is typically described in a polynomial manner (e.g., the uncanny valley). One reason for this pattern may be a higher sensitivity to deviations or errors in specialized categories like faces, bodies, or facial expressions. The present study found such a polynomial pattern in dynamic expressions of human and humanlike agents. However, this statistical pattern reverted to a linear relation when stimulus inversion disrupted specialized processing. Thus, specialized processing seems to drive changes in esthetic appeal across the human likeness dimension. Consequently, care must be taken in designing close to humanlike artificial entities, as even subtle errors or deviations can cause uncanniness.

## Data availability statement

The datasets presented in this study can be found in online repositories. The names of the repository/repositories and accession number(s) can be found at: https://osf.io/9cmhp/.

## Ethics statement

The studies involving humans were approved by the Ethics Committee of RIKEN, RIKEN. The studies were conducted in accordance with the local legislation and institutional requirements. The participants provided their written informed consent to participate in this study.

## Author contributions

AD contributed to conceptualization, investigation, formal analysis, data curation, drafting and revising the manuscript, and visualization. WS contributed to conceptualization, formal analysis, revising the manuscript, and visualization. C-TH contributed to validation and revising the manuscript. TM contributed to resources, supervision, and funding acquisition. All authors contributed to the article and approved the submitted version.

## Conflict of interest

The authors declare that the research was conducted in the absence of any commercial or financial relationships that could be construed as a potential conflict of interest.

## Publisher’s note

All claims expressed in this article are solely those of the authors and do not necessarily represent those of their affiliated organizations, or those of the publisher, the editors and the reviewers. Any product that may be evaluated in this article, or claim that may be made by its manufacturer, is not guaranteed or endorsed by the publisher.
